# Applications and Prospects of Agricultural Unmanned Aerial Vehicle Obstacle Avoidance Technology in China

**DOI:** 10.3390/s19030642

**Published:** 2019-02-03

**Authors:** Linlin Wang, Yubin Lan, Yali Zhang, Huihui Zhang, Muhammad Naveed Tahir, Shichao Ou, Xiaotao Liu, Pengchao Chen

**Affiliations:** 1 College of Engineering, South China Agricultural University, Guangzhou 510642, China; Wlinlin@stu.scau.edu.cn (L.W.); pengchao@stu.scau.edu.cn (P.C.); 2 National Center for International Collaboration Research on Precision Agricultural Aviation Pesticides Spraying Technology, Guangzhou 510641, China; 3 Department of Biological and Agricultural Engineering, Texas A&M University, College Station, TX 77845, USA; 4 USDA, United States Department of Agriculture, Agricultural Research Service, Water Management Research Unit, 2150 Center Ave, Building D, Suite 320, Fort Collins, CO 80526-8119, USA; huihui.zhang@ars.usda.gov; 5 Department of Agronomy, PMAS-Arid Agriculture University, Rawalpindi 46300, Pakistan; naveed@uaar.edu.pk; 6 Supernode Innovative Technology Co., Ltd, Shenzhen 518059, China; ou.shichao@supernode.cn (S.O.); liu.xiaotao@supernode.cn (X.L.)

**Keywords:** precision agriculture aviation, agricultural UAVs, obstacle avoidance, obstacle classification, obstacle avoidance zone, binocular vision

## Abstract

With the steady progress of China’s agricultural modernization, the demand for agricultural machinery for production is widely growing. Agricultural unmanned aerial vehicles (UAVs) are becoming a new force in the field of precision agricultural aviation in China. In those agricultural areas where ground-based machinery have difficulties in executing farming operations, agricultural UAVs have shown obvious advantages. With the development of precision agricultural aviation technology, one of the inevitable trends is to realize autonomous identification of obstacles and real-time obstacle avoidance (OA) for agricultural UAVs. However, the complex farmland environment and changing obstacles both increase the complexity of OA research. The objective of this paper is to introduce the development of agricultural UAV OA technology in China. It classifies the farmland obstacles in two ways and puts forward the OA zones and related avoidance tactics, which helps to improve the safety of aviation operations. This paper presents a comparative analysis of domestic applications of agricultural UAV OA technology, features, hotspot and future research directions. The agricultural UAV OA technology of China is still at an early development stage and many barriers still need to be overcome.

## 1. Introduction

China’s No.1 Central Document (jointly released by the Central Committee of the Communist Party of China and the State Council) has focused on agriculture, rural area and famer issues for 14 consecutive years since 2004. The Document clearly states that “we must strengthen agricultural aviation construction”, for which the development of modern agricultural plant-protection technology is of significance [1]. The Ministry of Science and Technology (MOST) and the Ministry of Agriculture (MOA, renamed the Ministry of Agriculture and Rural Affairs of the People’s Republic of China after March 2018) both regard agricultural aviation application as a supporting priority in “the 12th Five-year” scientific research plan, and the MOA approved a special scientific research on “Aviation Plant Protection of Staple Crops” in 2013 [2]. In the report of the 19th CPC National Congress [3], General Secretary Xi Jinping proposed “the strategy of rural vitalization” for the first time, insisting on prioritizing the development of agriculture and rural areas and accelerating their modernization.

With the steady progress of China’s agricultural modernization and the increasing demand for agricultural production, the agricultural aircrafts in our country have witnessed a rapid development. Agricultural UAVs, an important class of agricultural aircraft, have become a new force in the field of precision agricultural aviation. In 2016, the Department of Farm Mechanization of MOA carried out a special survey on the actual ownership and operating conditions of agricultural UAVs in 31 provinces (excluding Hong Kong SAR, Macao SAR and Taiwan) and in Xinjiang Construction Corps. The results showed that as of 30 June 2016, the number of field-operated plant-protection UAVs in the country was 4262, of which, electric multi-rotor agricultural UAVs were the mainstay. In 2015, their operation areas covered up to 476,035.67 hectares [1].

Due to their flexible operation, high efficiency, good control effect and economic benefits, agricultural UAVs are not only conducive to resource conservation and environmental friendliness, but they are also widely applicable to agricultural areas where ground-based machines have difficulty in cultivating [2,4,5,6,7]. Although it has shown prominent features and advantages in practice and application, as a new concept, their degree of Artificial Intelligence (AI) still needs to be improved and optimized in the aspects of safety factors, operation specifications, management mechanisms and data collection.

In the Joint Circular of the General Offices of MOA, the Ministry of Finance (MOF) and the Civil Aviation Administration of China on Launching Pilot Programs of Farm Machinery Purchase Subsidy for Standard Operation of Plant Protection Drone released in September 2017 [8], it was clearly specified for the first time that pilot drones should be equipped with an OA system so that the flight operation can identify, monitor and trace the surrounding environment. In March 2018, the circular of the MOA and the MOF continued to promote the agricultural UAV Purchase Subsidy (2018–2020) [9].

Benefit from breakthroughs in AI technology, parallel computing technology and intelligent hardware, agricultural UAVs’ OA technology is developing towards intellectualization, systematization and precision, while also transiting from the automatic piloting under known conditions of the past to real-time perception and intelligent decision-making regarding obstacles under unknown conditions so as to complete tasks independently. In the future, smart agricultural UAVs will greatly improve farming operations efficiency and play a core role in transforming and upgrading Chinese agricultural intelligent technology.

In order to implement the plans of “Made in China 2025” and “New Generation of AI Development Plan”, speed up the development of AI industry and promote the in-depth integration of AI and the real economy, the Ministry of Industry and Information Technology (MIIT) has issued the "Three-Year Action Plan for Promoting the Development of a New Generation of Artificial Intelligence Industry (2018–2020)” in December, 2017 [10].

Intelligent UAVs as mentioned in the Action Plan will support intelligent obstacle avoidance, automatic cruise, autonomous flight for complex environments, group engagements, and R&D of other key technologies and applications to promote new-generation communications, positioning, and navigation technology in UAV data transmission, link control, monitoring, and management applications, develop intelligent flight control systems, highly integrated specialized chips, and other key components. By 2020, intelligent consumer UAV 3-axis mechanical stabilization units should achieve a precision of 0.005 degrees, achieving 360-degree omnidirectional perception and realizing automatic and intelligent forced avoidance of air traffic control areas.

In addition, the Action Plan encourages local governments to increase investment and foster a group of leading enterprises in AI. Taking Guangdong Province as an example, in 2017, the scale of its AI core industry was about 26 billion yuan, and the civil UAV industry occupied a leading position in the world. DJI accounted for over 50% of the global market share of consumer UAV, and the output of civilian drones in Guangdong was 2,831,200, accounting for over 70% of the national market share. In August 2018, the Guangdong Provincial People’s Government stated in the notice on the issuance of New Generation AI Development Plan of Guangdong Province that it will build AI open innovation platforms, promote “Internet Plus” smart agriculture and strengthen R&D and industrialization of agriculture UAVs [11].

However, the farmland environment is complex and changing. During the outbreak of pests, in particular at night or low-light areas, if the UAVs mainly rely on pilots to observe and judge, the operations would be restricted and have high risk. Therefore, achieving autonomous recognition of obstacles and real-time avoidance is one of the inevitable trends in the intelligent development of agricultural drones.

OA technology of agricultural UAVs refers to the core intelligent technology whereby an agricultural drone can autonomously identify the obstacles types and complete the specified avoidance actions. The ideal OA system can automatically and promptly avoid all kinds of obstacles in the flight path, preventing accidents caused by operational errors, autonomous flight failures or other unexpected failures, and effectively reducing unnecessary loss of property and casualties.

## 2. Problems 

### 2.1. Complex Spraying Environment

Due to uncontrollable weather, like strong winds and floods, high temperatures and droughts, freezing damage and sandstorms, operation difficulty can increase dramatically. Taking the machine vision OA technology which is mainly affected by light intensity as an example, when there is specular reflection or other light pollution in the farmland, it can not only make drone pilots dizzy and uncomfortable, but also interferes with the recognition and judgment of the OA system due to the optical distortion and noise [12]. When sunlight is insufficient, the drone pilot’s vision will be weakened. In this case, the machine vision OA technology should be equipped with infrared technology to provide night vision ability. For instance, the XCOPE autonomous OA system produced by Guangzhou XAG (XAIRCRAFT) Co., Ltd. (Guangzhou, China) uses active near-infrared reflectance technology to realize night operations. Shenzhen DJI-Innovations Technology Co., Ltd. (Shenzhen, China) released its new Mavic 2 consumer drone in August 2018, and the addition of its Bottom Auxiliary Light that enhances the downward dual vision positioning system and allows it to land safely in low-light conditions. Besides, another drone, the Mavic 2 Enterprise released in October 2018, uses a dual spotlight to fly safer, and the latest T 16 agricultural UAV released in December 2018 also uses a safety searchlight to provide round-the-clock monitoring of spraying environments.

### 2.2. Multifarious Farmland Obstacles

During farmland operations, parameters such as flight velocity and altitude will directly affect the droplet deposition and pest control effects. The settings of those parameters are related to the type and growth of plants, topography and terrain of the operating area [13,14,15], etc. The safety of agricultural UAV operations should be ensured when precise low altitude and low volume spraying is implemented. Different production tools, such as plant protection nets, supports for climbing plants, as well as residential buildings, green spaces, power lines and towers, communication and lighting structures, and all kinds of living beings all increase the complexity of the OA environment and the need for OA technology. 

Due to the uncontrollable weather conditions and complicated farmland environment, the OA technology of agricultural drones needs to overcome the interference of illumination changes, scene rotation, low-resolution images [12], flight velocity and target occlusion to ensure safe operation under different external conditions. For instance, the visual OA sensor needs to avoid misrecognition caused by motion blur during high-speed operation, reduce lens distortion and improve imaging quality.

To solve the OA problem of agricultural UAVs and realize real-time perception, rapid image analysis, intelligent identification, potential areas detection, decision making of obstacle avoidance and other functions, it is necessary to fundamentally analyze the physical features of farmland obstacles, such as size, shape, type, etc., in a way to clearly identify OA parameters such as minimum recognition distance, OA operation commands, and response time. Therefore, two classifications will be provided based on the characteristics of various farmland obstacles that may appear in the operating environment. 

#### 2.2.1. Size-Based Classification

1. Micro obstacles: wire, inclined cable, branch, protruding plant, power grid or communication line, wooden pole or pergola placed in the field, test pole, nylon net, metal net, etc.
2. Small and medium obstacles: scattered tree, telegraph pole, hayrick, wind turbine, etc.
3. Large obstacles: shelter forest, high-pressure tower, house, meteorological tower, etc.
4. Non-fixed or visually-distorting obstacles: bird and beast, human being, high-speed moving non-living objects, blurring-texture or specular- reflecting objects, for instance, pond, plastic film in greenhouse, PC sun sheet, etc.

#### 2.2.2. Distance-Based Classification

The distance range can be adjusted based on actual operating conditions:

1. Short-range obstacles: within 5 m from the agricultural UAV.
2. Middle and long-range obstacles: 5 to 15 m from the agricultural UAV.
3. Long-range obstacles:15 m away from the agricultural UAV.

**Figure 1:**
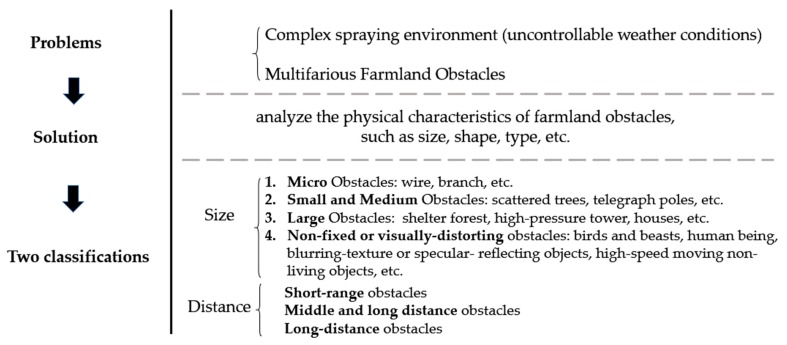
Analysis summary of farmland obstacles.

To summarize, these two classifications and the above content are also shown in Figure 1.

### 2.3. Obstacle Avoidance Zone

**Figure 2:**
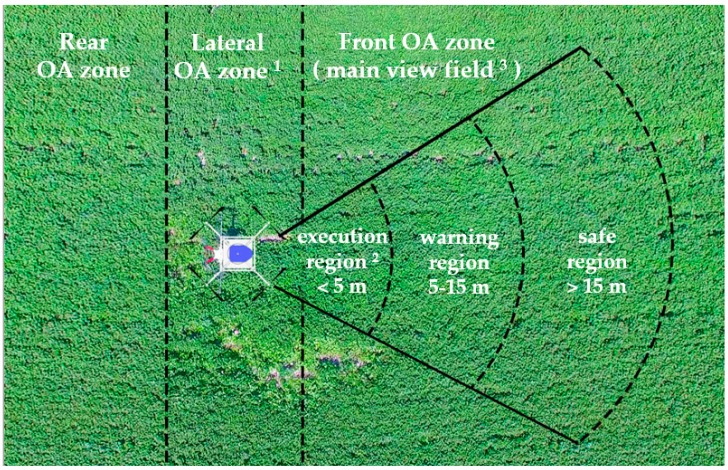
Obstacle avoidance zone of agricultural UAVs. ^1^ This figure does not express the OA zone in the up and down direction due to perspective. ^2^ The interval are subject to actual spaying environment and needs.^3^ The default main view filed is UAV’s heading direction.

For agricultural UAVs in flight, it is necessary to divide the view field of its main flight direction. Based on the obstacles detected by its own sensor system, different OA strategies are implemented for various obstacles in each area. We divide the front field of view into execution area, warning area and safe area, as shown in Figure 2.

Obstacle distance varies with the movement of the agricultural UAVs, and therefore it is relative. However, as Figure 1 shows, the OA zone of its main view field is fixed and absolute. In the execution area (<5 m), the OA motion commands shall be executed to avoid short-range obstacles; in the warning area (5–15 m), warnings shall be automatically performed for the medium-long-distance obstacles, and follow-up shall be carried out to prevent unexpected accidents; in the area (>15 m), obstacles can be ignored temporarily until they enter the warning area.

Because large, small and medium-sized obstacles have obvious features, they can be totally or partially detected by OA sensors in the safe area, when they enter the warning area or execution area, so it is relatively easy for drones to avoid them. The micro-sized and non-characteristic obstacles represent a small proportion of the field of view and they appear randomly and irregularly, so when they enter the warning area or even the execution area, it is still difficult to identify and avoid them successfully.

It is noteworthy that what occurs in the farmland mostly are micro-sized and non-characteristic obstacles. To evade such obstacles, the technical difficulties lay in how to achieve real-time perception of obstacles, rapid analysis and intelligent recognition of abundant images so as to quickly identify their potential areas. In line with the type and distance of the obstacles, the system needs to optimize OA paths and perform different OA actions involving numerous complex issues, such as the OA action response time and its implementation efficiency, adjustment of the flying speed, height and attitude of the drone, re-planning of flight path after obstacle avoidance, positioning signal loss prevention and anti-magnetic-field interference in the whole process, OA decision making of single, multiple, static or dynamic appearance of the obstacles.

At present, apart from improper manual operation or sudden failure of the machine, the main reason for most UAV crash accidents is OA failure, such as failing to avoid wires, branches and other small obstacles. Although the rate of crash accidents caused by small obstacles is relatively low, the property loss and casualty rates are extremely high. Therefore, how to avoid small obstacles should be the priority of agricultural UAV OA research.

## 3. Application of Agricultural UAV Obstacle Avoidance Technology in China

Currently, there are more than 200 plant-protection drone manufacturers in China, most of which are small and medium-size enterprises with weak technical strength and low level of R&D. Through investigation and searching on the National Business Administration System, we found that the manufacturers are distributed mainly in Guangdong (35 manufacturers), Shandong (24 manufacturers), Henan (11 manufacturers) and so on [1]. This section will take the actual production models of representative companies as examples, to analyze the current situation of domestic application of agricultural UAV OA technology.

### 3.1. Chinese Manufacturers of Plant-Protection Drones

Anyang Quanfeng Aviation Plant Protection Technology Co., Ltd. is an integrated service provider of agricultural intelligent vehicles. The company has completed the R&D, production and marketing of five agricultural drones with intellectual property rights, namely 3WQFDZ100-6, 3WQF120-12, 3WQF80-10, 3WQF125-16, 3WQFTX-10 and 3WQF294-35. AYQF is now planning to develop their own OA system.

Wuxi Hanhe Aviation Technology Co., Ltd. is currently offering the CD-15 pesticide spraying unmanned helicopter, and the Mercury-1 III and Venus-1 agricultural UAVs. Wuxi Hanhe currently provides an OA system as an option for users. For example, the CD-15 OA system uses “binocular + laser” to detect obstacles with a fixed radar underneath to achieve terrain-imitation flight.

The four-rotor 5 kg UAV JF01-04, six-rotor 10 kg UAV JF01-10 and eight-rotor 20 kg UAV JF01-20 are marketed by the Beijing VIGA UAV Technology Co., Ltd. VIGA provides two OA methods, namely a route OA system and a detection OA system. The former one, installed in each UAV, is a passive OA system dependent on route planning in advance to avoid known large and medium-sized obstacles in the operation field. The detection OA system, an optional accessory, can actively evade obstacles, but it has fewer users. VIGA has conducted OA tests on radar, infrared and visual sensors, and multi-sensor OA mode is regarded as a relatively mature method.

Guangzhou Tianxiang Aviation Technology Co., Ltd. (TXA) was established in 2010. With a new millimeter-wavelength radar OA system, its R-16 model adopts a two-way (front & rear) OA mode, which can effectively detect micro-obstacles such as wires and branches within a range of 30 m, which will effectively reduce the safety hazards in flight and greatly improve flight safety.

The M45, S40-E, M23-E and other plant-protection UAVs produced by Shenzhen GKXN Technology Co., Ltd. have been widely used in China. GKXN is developing a set of millimeter-wavelength radar OA systems that can perform real-time obstacle avoidance. So far, its OA systems have been placed on the front and rear of the unmanned S40-E helicopter and the multi-rotor UAV M23-E. The system can automatically detect obstacles in the front, like wires or branches with a diameter of 1.5 cm at a distance of 6 m. When encountering obstacles, the UAVs will hover autonomously and their forward-moving function will be locked until the danger is cleared. The bottom system can detect the changing terrain, then adjust the flight altitude and slope automatically. 

Guangzhou XAG Technology Co., Ltd.’s four models, the P20 2017, P10 2018, P20 2018, and P30 2018, are applicable to different farmland areas. The Xcope OA system can be equipped in forward-looking direction of the fuselage. The new generation of Xcope sensors can identify obstacles with a radius greater than 5 cm at a distance of 20 m, and conduct self-detouring to evade the obstacles. In addition, active near-infrared irradiation technology is used to give the UAVs night vision ability and ensure the safety of night operations. The newly launched 2018 drones are equipped with a millimeter-wavelength radar terrain-imitation module in a downward-looking direction to greatly improve its performance, replacing the 2017 ultrasonic terrain-imitation module. Now the detection range has expanded from no more than 4 m to 30 m, which can adapt to the complex and changing farmland landforms. The new P Series 2019 released in 14th December 2018 has been equipped with the latest industry-grade SUPERX®3 Pro RTK flight control system for precise navigation. With omnidirectional sensing capability, multiple redundancy system and AI fault prediction, it can automatically bypass farmland obstacles and ensure flight safety.

Shenzhen DJI-Innovations Technology Co., Ltd. entered the field of agricultural plant protection in December 2015. By the end of 2016, its MG-1S plant-protection UAV was launched. Compared with the FM-CW radar on the MG-1, the MG-1S is equipped with three high-accuracy millimeter-wavelength radars in the front, rear and beneath, respectively. Through non-stop scanning, the UAV can perceive the terrain changes in advance and adjust the flight height based on the topography and crop height to realize terrain-imitation flight. By the end of 2017, the newly released MG-1S Advanced and MG-1P equipped with the second generation high-accuracy radar have integrate the three previous directional radars and one OA radar, realizing the detection of wires with a radius of 0.5 cm 15 m ahead. The latest T16 released in 4th December 2018 has a reshaped overall structure and is equipped with a new DBF imaging radar, that can support two-way obstacle avoidance (front & rear). With digital beamforming technology, it can realize 3D point cloud imaging, which can effectively identify complex farmland scenes and achieve autonomous obstacle flight. The radar also supports terrain slope detection. The DJI non-agricultural UAVs use such OA systems as binocular vision, 3D sensing, infrared perception, ultrasonic OA modules, etc.

In the optional OA module market of China’s plant-protection UAVs, low purchase rates are a common phenomenon, because the relevant industries are not mature enough to cut the manufacturing costs and the existing active obstacle avoidance modules cannot fully meet customers’ needs.

We should also note however that: (a) the lack of definition or division of farmland obstacles; (b) the absence of technical performance indicators of OA system, such as response time, OA speed, etc.; (c) no unified and specific OA action decomposition and specification with only seemingly concrete but vague instructions such as hovering alarms, autonomous bypass, and emergency landing, all cannot meet the future requirements of independent spraying, real-time obstacle avoidance, continuous operation and cooperative work [16,17] of agricultural UAVs. This is also related to the lack of China’s precision aviation industry chain at present, and many fields are still in a blank or initial stage. In the near future, with the development and advances of science and technology, as well as the improvement of policies and regulations in various fields, the modernization process of China’s precision agriculture aviation will boost and soar up to the sky with long-term accumulation.

### 3.2. Application of Agricultural UAV OA Technology in China

Due to the complex farmland environment, various obstacles such as branches, wires and protruding plants are often encountered during operation. It is difficult for drone pilots to judge the surrounding flight environment when they are far away from the UAVs. Therefore, with the rapid development of science and technology, realizing autonomous identification of obstacles and real-time OA will be one of the inevitable trends in the intelligent development of agricultural drones. The current OA methods used in UAVs or unmanned ground vehicles (UGV) have their own advantages. What follows is the comparison of various OA technologies and the analysis of their applicability in the plant protection environment.

Real-time kinematic (RTK) positioning technology, as a common GPS measurement method, can obtain centimeter-level positioning accuracy quickly compared with single-point positioning technology. At present, RTK has been applied to agricultural UAVs and mapping UAVs. The agricultural UAVs equipped with RTK positioning systems can effectively avoid re-spraying, spraying leakage, and other problems caused by course deviation during the operation, and realize intelligent spraying (breakpoint continued spray or stopping spraying while evading obstacles) [18]. This saves labor costs and improves the efficiency and accuracy of spraying operations [16]. However, the RTK positioning technology has not been fully applied in agricultural drones because of its high cost, difficulty in deployment, and time-consuming and labor-intensive features. In addition to the existing RTK technology, XAG will open a RTK cloud network to allow users in remote mountainous areas to use RTK positioning service more quickly and get the operational maps of the land. The T16 UAVs of DJI have adopted a ”GNSS + RTK” dual redundant system to achieve centimeter-level positioning accuracy. RTK is more suitable for building obstacle maps of farmland than real-time obstacle avoidance. Large amounts of funds will be required for building RTK base stations and improving cloud technology.

Ultrasonic sensors have the advantages of simple structure, low cost, easy operation and it can be widely used in different media. Compared with other non-distance detection ranging methods, they have strong directivity and use less power. Not easily affected by light intensity, color variation and other factors, they can be used in poor environments such as dark, dust, smoke and so on [19]. However, the ultrasonic detection range is small, generally within 10 m, and the existence of the detection blind area limits the scope of ultrasonic ranging systems [20]. The emitted ultrasonic velocity is affected by ambient temperature, humidity and atmospheric pressure. In the atmospheric propagation, in addition to the losses caused by ultrasonic reflections and crosstalk between sound waves, there are additional attenuations caused by environment and other conditions, mainly including sound absorption, the influence of rain, snow and fog, and the ground effects of grassland, shrubbery and woods [21]. At present, ultrasonic sensors are mostly used as auxiliary safety devices, which are mainly applied to the down-vision system of agricultural UAVs, so as to get flight altitude parameters, achieve autonomous take-off and landing, or fly in complex terrains at very low altitude. For example, the crop protection drone P20 2017 of XAG is equipped with a waterproof ultrasonic sensor on the bottom of the fuselage to achieve high-precision terrain-imitation flight and prevent liquid drift.

Laser sensors are mainly used in autonomous navigation (laser gyroscope and laser homing) for military UAVs, because of their high precision when obtaining distance information, good directionality and strong anti-jamming ability. They require optical systems, making them unsuitable for high humidity, severe light pollution, dust, smoke and other environments, so they are rarely seen in agricultural drones [22,23]. Meanwhile, their production costs, volume and weight also make it difficult for them to meet the requirements of agricultural UAVs. Currently, laser scanning can only obtain discrete information of the scene, and with the scanning time, determined by the number of scanning points, it is difficult to achieve a good balance between real-time performance and range [24,25]. For example, 2D laser scanning can only acquire the depth information of the fixed angle in the front, and cannot obtain 3D depth information of the entire scene. Although 3D laser scanning can acquire 3D scene information, it takes such a long time to scan the entire scene [26] that the scanning speed can hardly be satisfactory for real-time obstacle avoidance.

Infrared sensing technology, a passive detection system, has strong anti-interference ability and good concealment. It can measure distances and describe contours at night or bad weather conditions [27,28,29]. However, the detection distance is small and the light emitted by the system is easily disturbed by the external environment. It must avoid the main energy band of sunlight [30,31] to evade interference or failure of the OA system, which is mainly caused by direct sunlight, reflections and the like. For this reason, it is mostly used in short-range sensing systems. Taking the consumer drone of DJI as an example, infrared sensors are mostly applied to identify the distance of the nearest objects from the drones, and monitor the surrounding environment to avoid collision when UAVs rise, descend or hover. For instance, the infrared barrier perception range on the top of the Inspire 2 is 0 to 5 m, and the maximum detection distance of the infrared perceptron on both sides of the Phantom4 Pro is 7 m. The upward and downward infrared sensing system on the Mavic 2, allows for a precision measurement range of up to 8 m. The aircraft hovers accurately at 50 m based on visual aids and lands safely by detecting land.

Structured light is emitted from the laser and converged into light bands of different shapes after passing through different lens structures. The linear light band is called line structured light. The laser and the lenses of various structures constitute the structured light sensor [32]. The line-structured optical sensor has the advantages of anti-interference ability, high precision, real-time performance and active control, so it is particularly suitable for robot measurement and control tasks in complex environments [33]. However, there are mutual interferences between adjacent structural light sensors. In outdoor environment, natural light almost nullifies structured light sensors. Therefore, the structured optical sensors are more commonly used in non-agricultural drones for indoor obstacle avoidance.

Time of Flight (TOF) ranging is one of the widely used ranging methods. It is a two-way ranging technology using the round-trip flight time of the measurement signal between the nodes to measure the distance. Compared with other ranging methods, it has the features of low energy consumption and easy deployment, and is suitable for applications where high ranging accuracy is required. The measurement signal is generally an electromagnetic wave signal and the propagation speed is close to the speed of light. Due to the transmission features of the light wave signal, non-linear propagation factors such as reflection, refraction and diffraction can all cause measurement time deviations, which will lead to huge distance calculation errors [34,35]. Hence, TOF ranging can be highly accurate in an open environment, but can generally produce a large number of errors in complex environments. When applied in agricultural drones, safety auxiliary device derived from the combination of TOF ranging principle and other sensors is more suitable for obstacle avoidance.

Millimeter-wavelength radar is a detection radar working in the microwave band. Its frequency domain is 30 to 300 GHz [36]. Due to the complex farmland operating environment, ultrasonic and other sensors based on optical principles are easily affected by climatic conditions, whereas millimeter-wavelength radars can work at all weather conditions with their strong penetrating ability, large operating distance, reliable detection and anti-electromagnetic interference [37,38,39]. Millimeter-wavelength radar has been widely used in vehicle obstacle avoidance. For instance, automatic cruise navigation, forward-backward collision prevention systems, blind spot detection systems and Blind Spot Information system (BLIS) are all equipped with millimeter-wavelength radar to detect environment around the car body. However, the car driving environment is a 2D scene, whereas the obstacle avoidance environment for agricultural drones involves the complex 3D farmland environment. Compared with other ranging sensors, the millimeter-wavelength radar has low resolution and high cost. It can only detect parallel distances but can hardly describe the outline of the OA objects as well as their angle in the field of view [40,41]. Its short-range detection capability can be replaced by the ultrasonic, laser, infrared and other obstacle avoidance range sensors mentioned above. Therefore, millimeter-wave radar is currently mainly used in terrain-imitation flight systems for agricultural UAVs. For example, the 2018 new models of XAG all install millimeter-wave radars as terrain-imitation modules, enabling the drones to work in the complex and ever-changing farmland. Its maximum detection range is up to 30 m. Besides, the DJI MG-1S plant protection drone is equipped with high-precision millimeter-wave radars in the front, rear and below. The front and rear radars can detect the terrain in advance and work with the below radar to accurately determine the flight height. The MG-1S can also perceive obstacles in the range of 1.5 to 30 m with its millimeter-wavelength radar. When an obstacle is detected, it will hover automatically to prompt the pilots and suspend the work until danger passes.

In visual obstacle avoidance, monocular visual ranging mostly uses the measurement method based on given motion, which is to use the moving information and the images captured by the camera to measure the depth distance [42,43,44]. It is simple in structure, mature in technology and fast in computing speed [45], but it cannot directly obtain the depth information of obstacles. Measurements using a single feature point are error-prone due to inaccuracies in feature point extraction. When matching a feature point of one or more pictures, the matching error has a significant effect on the measurement results. Meanwhile, the processing time rises with the increase of pictures [44]. Therefore, monocular vision ranging needs to be photographed in a well-lit environment, and the images should be of high resolution and clear texture so that they can be effectively processed [46]. Given the facts of the complex operating environment, large amount of information and the increasing calculation of algorithm, monocular ranging is difficult to meet the requirements of real-time performance and accuracy of obstacle avoidance for UAVs. A single vision system is installed on the left and right sides of the MAVIC 2, which is capable of detecting obstacles at speeds of up to 28.8 kph and assisting in obstacle avoidance.

Drawing on the ability of human eyes to perceive 3D space, binocular stereo vision technology gets the parallax result through binocular image acquisition, image correction, stereo matching and other steps. Then the depth information of the scene is calculated to reconstruct the 3D information of the space scene. Featuring good concealment and the ability to obtain comprehensive information (including color and texture of the obstacles), as well as the 3D depth information of the scene [7,47,48,49,50,51,52], the stereo vision technology can recognize and measure the distance between the fuselage and the obstacles. However, the most critical problem of binocular vision lies in stereo matching. The effects of illumination changes, scene rotation, object occlusion, low image resolution, interference or even overwhelming of target features all lead to instability of the target features and degraded accuracy of object detection. Meanwhile, there is still lack of stereo matching algorithms that can effectively balance the binocular matching accuracy and operation speed. [7,46]. At present, binocular stereo vision technology is more used in consumer and professional UAVs, such as the Mavic series, Phantom 4 series and Inspire 2 of DJI, the upcoming Xplorer 2 model of Shenzhen Zero UAV Tech. Co., Ltd. (Zero), the TYPHOON H and H520 of Yuneec International Co. Ltd. They all use binocular stereo vision to achieve obstacle perception and automatic obstacle avoidance. The forward dual vision sensors of Mavic 2 allow for a precision measurement range of up to 20 m and its detection range is 20–40 m, which is capable of detecting obstacles and timely stops when flying at speeds of up to 50.4 kph. Compared to the forward one, the measurement and detectable range of its downward and backward dual vision system are shorter. For agricultural UAVs, the CD-15 plant-protection unmanned helicopter by Wuxi Hanhe Aviation Technology Co., Ltd. provides a “front and rear binocular + laser” OA system as an option for users. P30 2018 plant-protection drone of XAG is equipped with XCOPE autonomous OA system, enabling it to sense the forward environment, identify obstacles with a radius of more than 5 cm within 20 m and bypass them autonomously, but smaller obstacles such as wires still cannot be recognized in real time.

Table 1 shows the comparison and analysis of the OA sensors mentioned above to show whether they are suitable for agricultural drones to avoid obstacles or not.

## 4. Analysis and Prospects

By analyzing the application status of domestic OA technologies for agricultural UAVs and the comparison of each OA sensor, it can be seen that, different OA sensors are suitable for different plant-protection environments and OA distances, the future research hotspots of Chinese agricultural UAVs’ OA technology will focus on the following three aspects:

### 4.1. Multi-Sensor Obstacle Avoidance Forms

With the development of precision agricultural aviation technology, multi-sensor OA system [53,54] will become the mainstream trend of real-time OA system for Chinese agricultural UAVs. The integration of visual and non-visual sensors will also improve the safety of plant protection operations, providing many possibilities for autonomous spraying and intelligent navigation. Therefore, we consider using multiple OA sensors to achieve an ideal OA effect.

### 4.2. Real-Time Active Obstacle Avoidance Technology

At present, agricultural UAVs are widely used in the agricultural areas where ground-based machinery have difficulty in cultivating. However, the method of calibrating obstacles in advance to obtain a safe flight path or the method of constructing a temporary observatory for pilots is not suitable for large-area farmland where farming conditions are changing rapidly or plant protection staff can’t go deep. Based on the severe situation of the continuous shortage of agricultural labor force in China, the realization of all-weather autonomous operation will be one of the development trends of future agricultural UAV. The achievement of autonomous spraying, continuous operation and collaborative work is inseparable from breakthroughs in OA technology and endurance capacity. The realization of real-time active OA technology will greatly improve the safety and intelligence degree of agricultural UAV farmland operations, provide protection for the smooth implementation of continuous operation of variable spraying [55], and play an important role in promoting the development of precision agricultural aviation application and OA technology.

### 4.3. Development of OA Auxiliary System & Standardization of OA Process

Due to the farmland’s complex operating environment and the limitation of different sensor applicable environment, it is inevitable that the OA sensor cannot directly detect the micro-obstacles or only obtain discontinuous or scattered information of the obstacle fragments. If it only focuses on the identification of obstacles themselves, it will be a big technically difficulty and hard to achieve maturity in a short time. Therefore, we can consider indirect identification methods, for example, identifying an alternative of micro-small obstacles, using it as an OA auxiliary method for agricultural UAV, and establishing a feature database of farmland obstacles. This method mainly focuses on identifying micro-obstacles, supplemented by their alternatives to achieve effective obstacle avoidance, for example, the wire substitute is its wire pole (or tower), and the replacement for branches is the tree crown. What is mostly found in China’s farmland are 10 kV high-voltage cables, which generally rely on the installation of poles at certain intervals to withstand the weight of wires and balance the external forces. Wire towers may appear in the farmland, but obviously, they are much easier to identify than utility poles. 

**Figure 3:**
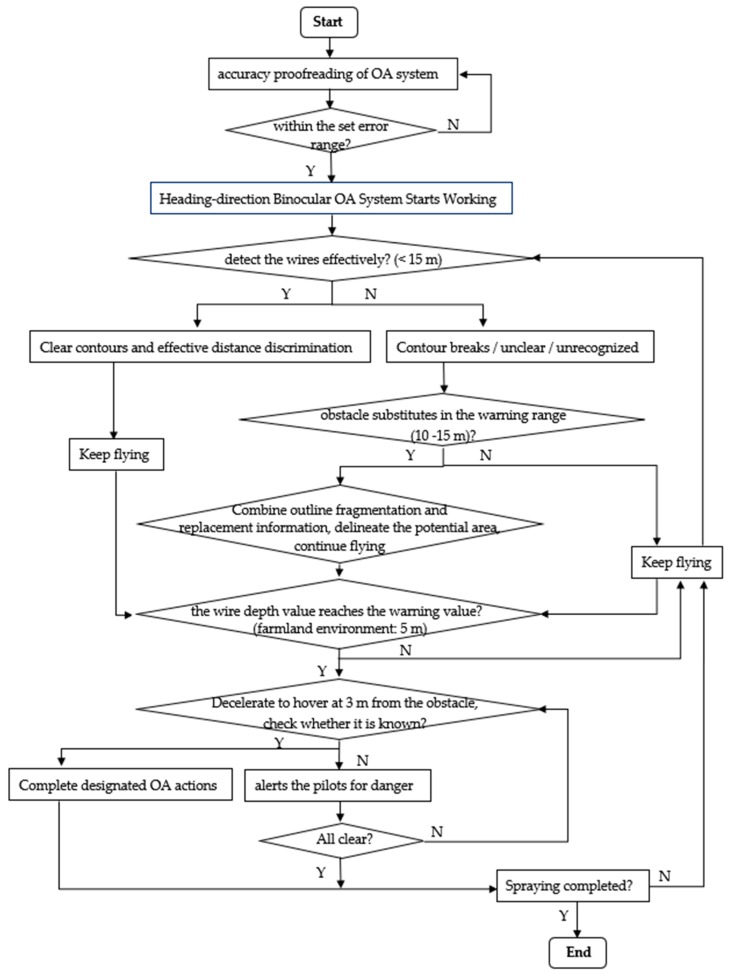
Obstacle avoidance flow chart of micro obstacles for Agricultural UAV based on binocular vision sensor.

Considering the obstacle avoidance zones and obstacle classification in Section 2, taking binocular vision sensor as an example and selecting the micro-sized obstacle high-voltage wire as target, its OA flow chart is shown in Figure 3. It is worth noting that this OA method is independent of the specific sensor category, and if non-visual sensors can achieve the same detection effect, they are also applicable to this process. When the outline of the wire detected by the OA system is clear and the distance can be effectively determined, the corresponding OA instruction can be directly executed according to its depth information. This kind of situation belongs to direct recognition, which is an ideal OA situation. When the direct recognition fails, which means the system only get some broken outlines, unclear fragments, or no recognition at all, it can identify indirectly small obstacle substitutes with large area, volume or density in the view field (e.g., the replacement of wires is telegraph poles), and combine the fragment information of small obstacles (e.g., incomplete wire contour fragments), the missing obstacle information can be supplemented, and the potential region of obstacles can be restored. Finally, combined with depth information, different OA instructions are executed according to different OA zones. This indirect method can use the original OA sensor to develop obstacle avoidance assistance system based on the original load capacity, and reduce the impact of misidentification and misjudgment during operations when direct identification fails. It provides a new idea for the research on obstacle avoidance of agricultural UAV. The standardization of OA process is conducive to the standardization of OA action, which will help to obtain the best flight order when multiple agricultural UAVs cooperate in the future, and promote production efficiency. This standardization research will promote the establishment of OA technology and help agricultural drones to achieve safe operation and successful obstacle avoidance.

## 5. Conclusions

With the promotion of the Pilot Programs of Farm Machinery Purchase Subsidy for Standard Operation of Plant Protection Drone, the urgency of further research on the OA technology of agricultural UAVs cannot be ignored. Deepening the understanding of the emerging OA technology will provide a better OA scheme for agricultural drones, and provide references for the orderly development of China’s and even the world’s agricultural UAV OA technology. Breakthroughs in theoretical research and related equipment R&D will greatly promote the rapid development and application of precision agricultural aviation OA technology in China, and truly achieve high-efficiency, green and safe plant protection requirements.

## Figures and Tables

**Table 1:** Comparison of Various Obstacle Avoidance Sensors.

Sensor Type	Max Range/m	Advantages	Disadvantages	Whether Suitable for Obstacle Avoidance of Agricultural UAV	Applied Agricultural UAVs/Manufacturers
RTK	----	accurate	no shelter	on-site calibration, suitable for creating obstacle maps, not for real-time OA	XAG: P20/30 2018 P series 2019 GKXN: M23-E, S40-E Hanhe: Venus-1 AYQF: 3WQFTX-10 DJI: T16
Ultrasonic sensor	<10	cheap	near detection distance, a blind spot for detection, vulnerable to environment	low resolution, more suitable for short-range OA safety auxiliary device	XAG: P20 2017
Laser/initiative infrared sensor	<50	high resolution, reliability	required mechanical, scanning, single point measurement is unreliable, multi-wire solid state sensor is expensive and immature	High requirements on environment, only acquire discrete information, suitable for short-distance obstacle avoidance	DJI (consumer UAVs): Inspire 2, Mavic 2, Phantom 4 Pro Hanhe: CD-15
Structured light sensor	<10	high resolution, more reliable than binocular	adjacent structure light sensors interfere with each other, vulnerable to outdoor natural light	only suitable for indoor OA	----
TOF	<10	high reliability	low resolution, susceptible to environmental interference	small sensing range, suitable for OA auxiliary device	----
Millimeter-wave radar	<250	high reliability, can be tested in heavy rain, dense fog and others	low resolution, high cost	More applied to agricultural UAV terrain-imitation flight, the OA/anti- collision system with middle and long distance is cost-ineffective	XAG: P series 2018/2019 DJI: MG-1S GKXN: S40-E TXA: R-16
Monocular vision	<10	low cost, less demand of computing resources, relatively simple system architecture	continually update to maintain a large sample database	Low reliability, more suitable for static or 1D moving objects.	DJI (consumer UAVs): Mavic 2
Binocular vision	<100	high resolution	needing adequate lighting	change the baseline to detect obstacles at different distances, suitable for farmland OA problem	XAG: P20 2017, P20/30 2018 P series 2019 Hanhe: CD-15, Mercury-1 DJI: consumer UAVs
